# Incidence of multiple sclerosis in China: A nationwide hospital-based study

**DOI:** 10.1016/j.lanwpc.2020.100010

**Published:** 2020-08-06

**Authors:** De-Cai Tian, Chengyi Zhang, Meng Yuan, Xin Yang, Hongqiu Gu, Zixiao Li, Yongjun Wang, Fu-Dong Shi

**Affiliations:** a China National Clinical Research Center for Neurological Diseases, Beijing Tiantan Hospital, Capital Medical University, Beijing 100070, China; b Department of Neurology, Tianjin Medical University General Hospital, Tianjin 300052, China

**Keywords:** Multiple sclerosis, Incidence, Hospitalization burden, National study

## Abstract

**Background:**

Multiple sclerosis (MS) is a leading cause of disability among young adults and effects considerable social and economic burdens. Data of MS incidence in China at the national level is lacking. We conducted the first nationwide hospital-based study to estimate the incidence and hospitalization burden of MS in China.

**Methods:**

This study is based on an administrative database of the National Hospital Quality Monitoring System, which covers all 1665 tertiary hospitals in mainland China. The “Medical Record Homepage” of all patients, including 346 variables including demographic characteristics, diagnoses, procedures, and expenses etc., were uniformly collected across each tertiary hospital via standard protocol. MS was defined by the 2010 International Panel criteria for MS and was identified by ICD-10 code (G35•0).

**Findings:**

We identified 27,336 hospital admissions for 15,060 MS patients from 2016 to 2018; amongst these patients, 9,879 were newly diagnosed. The age- and sex-adjusted incidence per 100,000 person-years is 0•235 (95% confidence interval [CI] 0•230–0•240), with 0•055 (0•050–0•060) in children and 0•288 (0•282–0•294) in adults, respectively. The female to male ratio is 2•02; the peak disease onset is age of 40–49 years. Residents in high-latitude and high-altitude areas are more likely to develop MS (F = 8•99; p < 0•001). Prevalent comorbidities include hypertension (18•8%), diabetes (7•2%), stroke (14•7%), depression or anxiety (3•7%), and autoimmune disease (2•3%). Through 2016–2018, 104 adults and 2 children died, with a hospital mortality rate of 9•9 per 1,000 person-years.

**Interpretation:**

For the first time, we obtain the national incidence of MS as 0.055 in children and 0.288 in adults per 100,000 in China. The geographical distribution of MS incidence presented a north-south latitude gradient and a west-east altitude gradient.

**Funding:**

National Science Foundation of China (81801199, 91642205, and 81830038); Advanced Innovation Center for Human Brain Protection, Capital Medical University, Beijing.

**Research in context**

**Evidence before this study**

A systematic search of PubMed and the web of science was conducted from 1985 up to 2020, with terms defined as “multiple sclerosis” AND (“prevalence” OR “incidence” OR “epidemiology”) AND (“China” OR “Chinese” OR “Hong Kong” OR “Taiwan”). Two systemic reviews and eleven articles were identified. After removing duplicates, there were five related articles for Taiwan, four for Hong Kong, and four for Mainland China. From these regional studies, we extrapolated that the prevalence of MS ranges from 1•39 (Shandong) to 5•2 (Shanghai) per 100,000 persons in mainland China throughout 2008 to 2013, 0•77 to 4•8 per 100,000 persons in Hong Kong during 1999 to 2008, and 0•84 to 4•99 per 100,000 persons in Taiwan during 1975 to 2009. The annual incidence was 0•12 per 100,000 in males and 0•2 per 100,000 in females in Shandong province in 2013, while it was 0•448 to 0•83 per 100,000 between 1997 and 2008 in Taiwan. Collectively, studies on MS incidence in China is sparse and currently limited at the regional level.

According to these studies, the mean onset age ranged from 25•9 to 46•4 with a downward trend and the female to male ratio was 1•2 to 9•6. The mortality rate has fallen from 9•3% to 7•1% in Taiwan within about a decade.

**Added value of this study**

This is the first-ever nationwide survey of MS in mainland China. We estimated the age- and sex-adjusted incidence of MS across all age groups at 0•235 per 100,000 persons and compile a map of the incidence rates in 31 provinces and municipalities in mainland China. China's vast territory encompasses a broad latitude range (20°N to 50°N), in the northern hemisphere, that can be broadly arranged into three macro landform complexes, descending step- by-step from the Qinghai-Tibet Plateau to the coastal area in the east. This three-step “staircase” presents an opportune model for analyzing the geographic distribution of multiple sclerosis. The present study confirms a negative west-east altitude gradient for disease distribution and further reports a negative north-south latitude gradient for disease distribution. As the latest government effort to reform China's health care system, the Urban Resident Basic Medical Insurance and New Rural Cooperative Medical Insurance covered about 70% of all patients, and the hospitalization burden displayed a downward trend. Comorbidities and mortality of MS were also evaluated. The ratio of MS and neuromyelitis optica spectrum disorders (NMOSD) is 1•0: 1•21.

**Implication of all the available evidence**

The MS incidence rate in China is 0•235 per 100,000 person-years, which is comparable with East Asian countries. A west-east gradient of altitude for incidence is identified, in addition to north–south latitude gradient. In our study, young adults are more likely to develop MS at high-latitudes or high-altitude regions. The incidence rate of MS and NMOSD is comparable in China. This national administrative database enhances the accuracy of estimates for policy makers, providers, and multiple sclerosis societies to improve health-service planning. The reported disease burden calls for dramatically increased regional and global efforts in MS patient care as well as investment in research for this devastating disease.

Alt-text: Unlabelled box

## 1 Introduction

Multiple sclerosis (MS) is a classical immune mediated demyelinating disease of the central nervous system, and it is a leading cause of disability amongst young adults worldwide, affecting considerable social and economic burden [1]. Nearly 1 million MS patients were confirmed with associated costs of over $24 billion annually in the United States [2]. In 2020, Public Health England revealed about 4950 people are diagnosed with MS each year in United Kingdom [3].

MS Atlas, as compiled by the MS International Federation estimated a global median prevalence rate of 35/100,000 and a median incidence rate of 4 per 100,000 [4]. However, the worldwide prevalence of MS varies substantially between the continents due to geographical and environmental characteristics. The landmark epidemiological studies from Kurtzke and colleagues assumed the frequency of MS was related to latitude with three; high, moderate, and low “zones” [5]. The highest age-standardized MS prevalence was 164•6 in high-income North America, 127 in western Europe, and 91•1 in Australasia, and the lowest were 3•3 in eastern sub-Saharan Africa, 2•8 in central sub-Saharan African, and in Oceania, 2 per 100,000 persons [6].

Asians have a lower risk of MS as the prevalence estimates in Asians are amongst the lowest in the world [7]. The nationwide Japanese survey conducted in 2004 estimated the prevalence of MS at 7•7 per 100,000 [8]. The age-standardized prevalence per 100,000 persons was 3•23 in Korea [9] and 2•73 in Malaysia [10]. However, in China, which accounts for 21•6% of Asia's land area and 18•8% of the world's population, the epidemiological data on MS is absent or extremely sparse. The two epidemiological studies available are regionally narrow; one showed the MS prevalence rate of 1•39 per 100,000 in Shanghai, the other in Shandong Province estimated an incidence rate of 0•20 per 100,000 for females and 0•12 for males [11,12].

In this study, we estimate the incidence of MS in mainland China between 2016 and 2018 using a nationwide administrative database of Hospital Quality Monitoring System (HQMS), which is maintained by the National Health Commission (NHC).

## 2 Methods

### 2.1 Study design

This was a population-based retrospective study based on a national database of the Hospital Quality Monitoring System (HQMS), which covers the entire Chinese population. The present study was a project of China National Clinical Research Center for Neurological Diseases and China National Center for Quality Control of Neurological Diseases and was approved by the Institutional Review Board at Beijing Tiantan Hospital.

### 2.2 Data sources and collection

China is an East Asian country encompassing latitude range of 18°24 N to 52°33 N, and as of 2018 houses a population of 1•39,538 billion, according to the China National Bureau of Statistics where geographic and demographic information were derived from (http://www.stats.gov.cn). The Chinese can be considered a relatively homogenous population; overall, 91•51% are Han Chinese and 55 other ethnic groups represent the remaining portion. The HQMS was launched in 2011 and covers all tertiary hospitals of 31 provinces and municipalities comprising mainland China. As part of China's health system reform, the HQMS is designed to monitor the quality of medical care to conduct the performance appraisal of public hospitals. The system consistently collects a dataset of information from all inpatient medical records across each tertiary hospital via a standard protocol, including demographic characteristics, diagnoses and in-hospital mortality procedures, expenses, etc. This unique nationwide patient identification system allows linkage across time and distance barriers, taking into account the country's large size and population mobility (appendix).

**Fig. 1:**
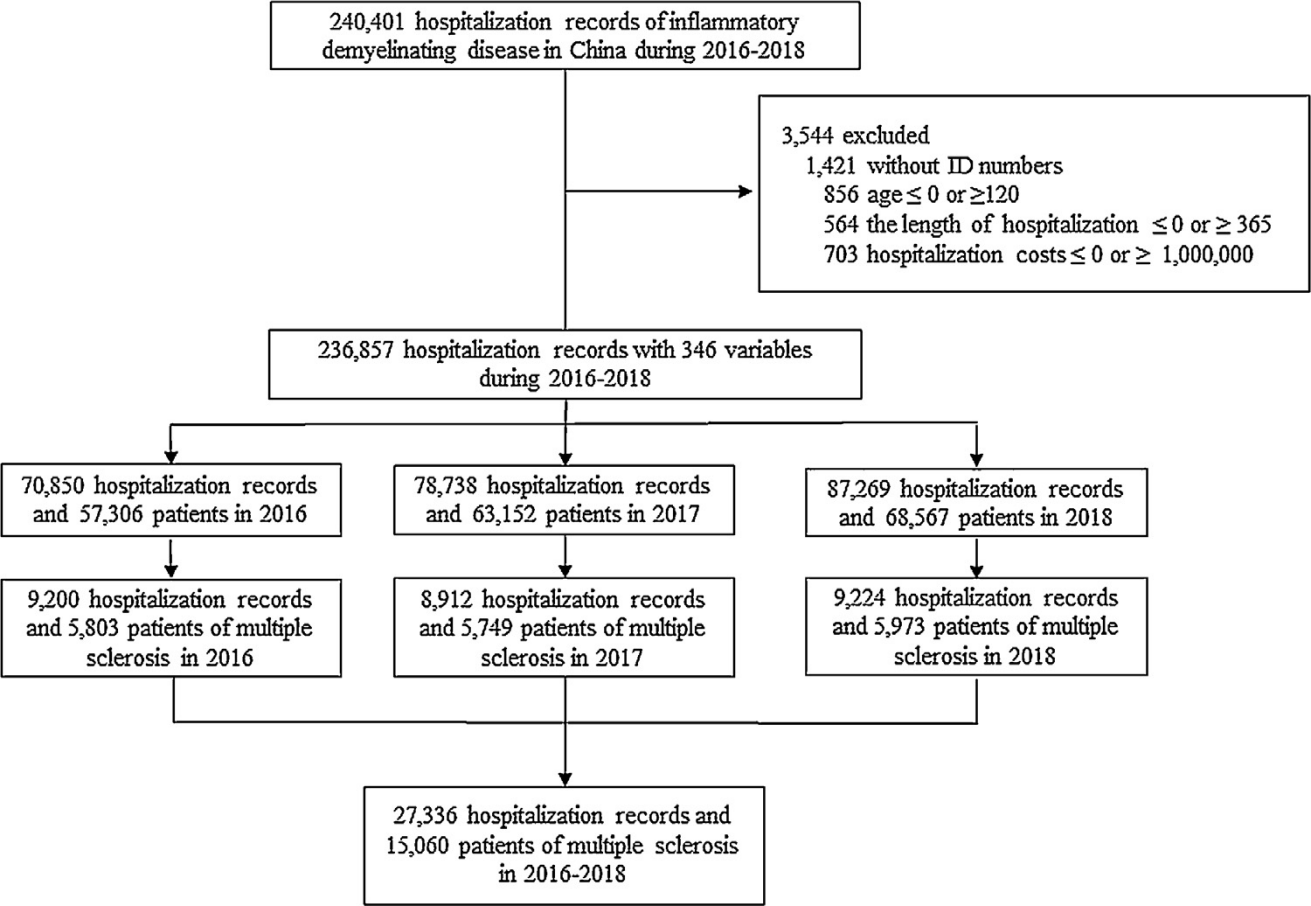
The incidence of multiple sclerosis in China: flowchart of study population selection.

According to the China Health Statistics Yearbook 2018, there are 2340 tertiary hospitals in China. The 1665 hospitals surveyed in this study covered 98•5 percent of tertiary public hospitals, where the vast majority MS patients are diagnosed and managed. The remaining 228 private hospitals and 422 traditional Chinese medicine hospitals rarely accommodate these patients. The 240,401 hospitalization records were retrieved from the HQMS database between 1^st^, January 2016 to 31^st^, December 2018, based on the diagnosis of inflammatory demyelinating disease (Fig. 1). Hospitalization records were excluded for those without ID numbers, ages < 0 or ≥120, length of hospitalization ≤ 0 or ≥ 365 days, and hospitalization costs ≤ 0 or ≥1000,000. "The same person" individual identification parameter is generated in the database based on name, gender, and Citizen Identification Number (a unique, unchanging legal number). This individual unique patient label within the database avoids duplication of a patient at different hospitals or departments across the country.

### 2.3 Case ascertainment

MS patients were identified based on the International Classification of Diseases, Tenth Revision (ICD-10) code G35.0 in principle or other discharge diagnoses. The diagnoses of multiple sclerosis were based on the 2010 revised McDonald criteria [13]. HQMS requires a “Quality Assurance Physician” and coder for each uploaded record, to review the diagnosis accordance of ICD-10 code. Patients who were initially discharged with a principle diagnosis of MS were defined as “new-onset cases”. New-onset patients in each province or municipality were determined based on "from province" HQMS parameter. An inherent advantage of HQMS structure is its feature which includes all MS patients from all departments (emergency, neurology, pediatrics, ophthalmology or etc.). Protocol details are provided in full in the appendix. In alignment with WHO guidelines, this study defines an adult is a person older than 19 years of age, and a child is a person 19 years or younger.

### 2.4 Statistical analysis

We estimated the annual incidence in each province and municipality by dividing the number of cases by the local populations from 2016 to 2018. Incidence was categorized by sex and analyzed across age groups by 5-year increments. Population data from the 2010 Chinese Census was used as reference to calculate incidence rates adjusted for age and sex (http://www.stats.gov.cn/). 95% confidence intervals (CI) were calculated by the Poisson distribution. Using the Spatial Statistics Tools, we analyzed the distinctive features of spatial distributions and identify statistically significant spatial clusters or spatial outliers. We also evaluate the overall pattern of clustered/dispersed and establish a spatial relationship model. The globe Moran's I index represents the global incidence spatial autocorrelation and distribution pattern. Here latitude is divided into high, medium and low zones which corresponds to latitude 40°–50°, 30°–40°, and 20°–30°, respectively. The three-step “staircase” topography is delineated by regions divided into three altitude gradients 500 m, 1000–2000 m, and >4000 m. Multiple Linear Regression was used to analyze the impact of altitude and latitude of MS incidence. Age of diagnosis and length of hospital stay were calculated and expressed as mean ± standard deviation (SD). Hospitalization costs are presented as median with range (25^th^ and 75^th^ percentiles). Number and proportion of patients were catalogued for comorbidities and payment methods. Deaths from MS in the hospital were analyzed by the proportion of the discharge department and underlying causes. The incidence distribution map was drawn through ArcMap 10•7. All the analyses were performed with SAS software (version 9•4, SAS Institute Inc., Cary, NC, USA). Details of protocol are included in the appendix section 3.

### 2.5 Role of the funding source

The funding sources of this study did not influence nor participate in study design, data collection, data analysis, data interpretation, or drafting of the report. The corresponding author had full access to all the data in the study as well as final responsibility for the decision to submit for publication.

## 3 Results

### 3.1 Incidence of MS

**Fig. 2:**
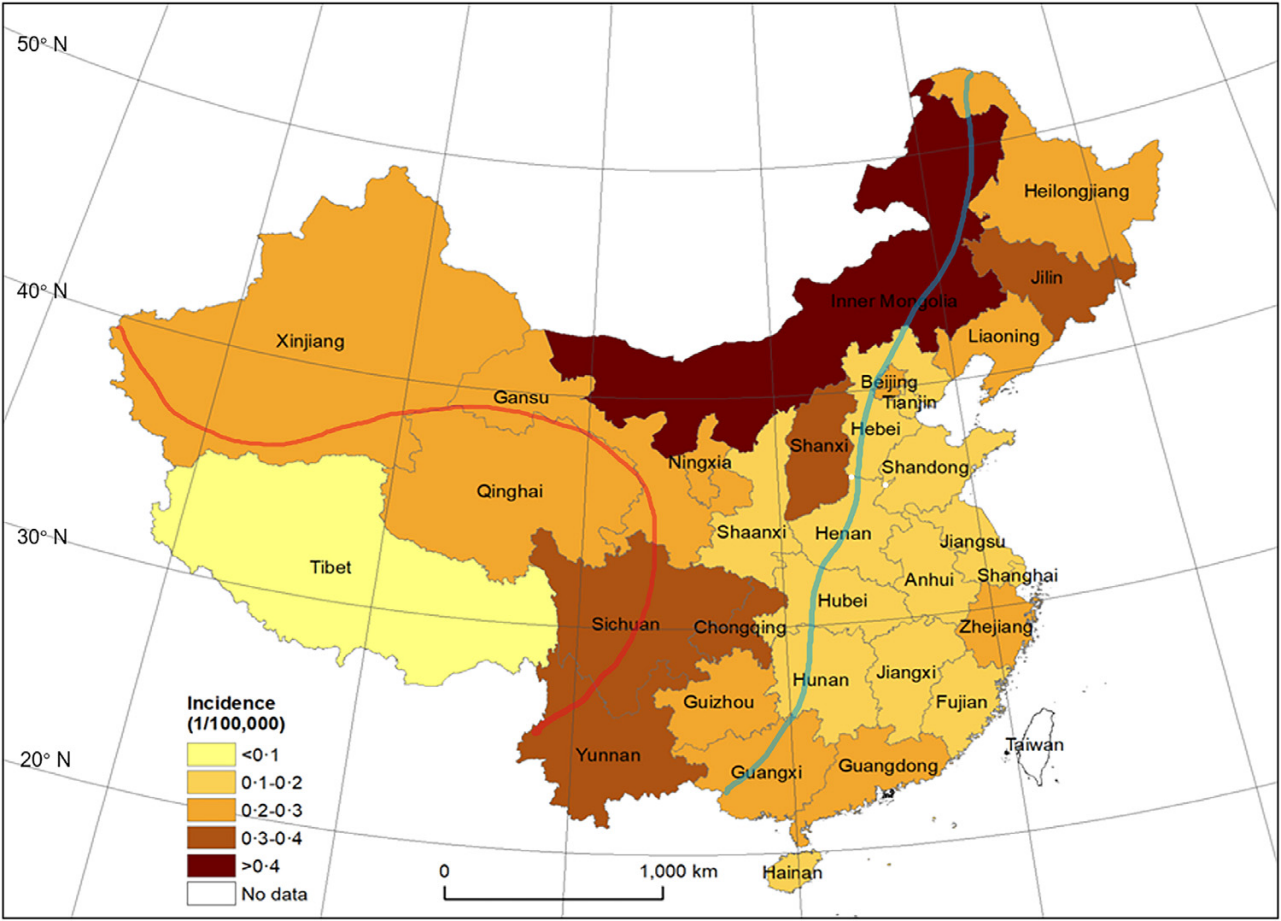
Incidence map of MS in mainland China, 2016–2018. The study was conducted in mainland China. Hong Kong and Macao not included. In mainland China, there are three latitude gradients from north to south: high latitude (40°–50°N), mid-latitude (30°–40°N) and low latitude (20°–30°N) zone. The land surface of China slopes down from west to east in a three-step staircase. The three altitude gradients (<500 m, 1000–2000 m, and >4000 m) are indicated with blue and red dividing lines, respectively.

27,336 hospital admissions from 15,060 MS patients in 2016 to 2018 were identified for evaluation (Fig. 1). Amongst these patients, 9879 people with MS were newly diagnosed: 3917 in 2016, 3107 in 2017, and 2855 in 2018. The age- and sex-adjusted incidence of MS in Chinese was estimated as 0•235 (95% confidence interval [CI] 0•230–0•240) per 100,000 person years during the study period. The estimated crude incidence in the context of geographic distribution varied from 0•049 (95% CI 0•006–0•093) in Tibet (30° N) to 0•541 (95% CI 0•488–0•593) in Inner Mongolia (40° N) (Fig. 1). The global spatial correlation analysis of MS incidence data for 2016–2018 shows that the distribution of MS incidence is dispersed rather than randomly distributed (Moran's I > 0, P > 0.05, Z < 1.96). Inner Mongolia was a high incidence cluster from 2016–2018; Guangdong was a high-incidence gathering place in 2016; Jiangxi, Fujian, Jiangsu and shanghai were low-value gathering places in 2016, and Anhui and Shanghai were low-value gathering places in 2017 (appendix). The risk of MS is lower in mid-latitudes (β = -0•203, standard error (SE) = 0•05, p < 0•05) and low-latitudes (β = -0•135, SE = 0•05, p < 0•05) compared to high-latitudes. The incidence of the low-altitude eastern regions compared with the high-altitude western regions, find that residents in lower-altitude areas were less likely to develop MS (β = -0•145, standard error (SE) = 0•04, p < 0•01) (Fig. 2).

The age- and sex-adjusted incidence of adults was significantly higher than that of children (p < 0•001), with 0•288 (95%CI 0•282–0•294) in adults and 0•055 (95% CI 0•050–0•060) in children. The proportion of patients under 19 years old was less than 5%; peak incidence occurred in those aged 40–44 and 45–49 years, with 0•356 (95% CI 0•335–0•377) to 0•360 (95% CI 0•341–0•380), respectively.

### 3.2 Ratio of MS and NMOSD in China

**Fig. 3:**
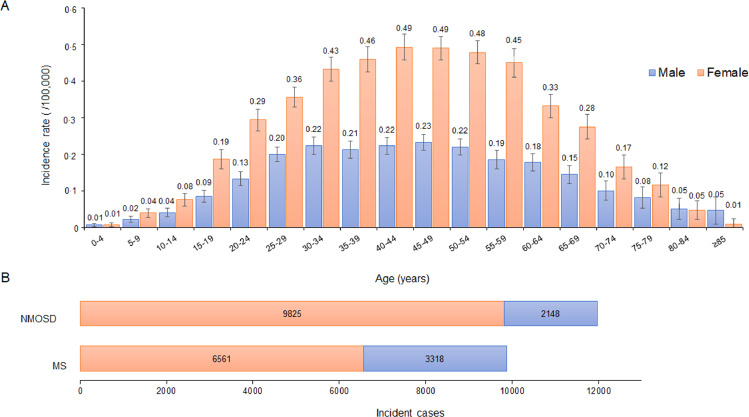
The incidence of MS in the all age groups (A) and Comparison of MS and NMOSD (B) (A) Depicts the crude incidence rate of MS in all age groups in males and females during 2016–2018. The number of all MS and NMOSD incident cases between 2016 and 2018 is illustrated in men and women (B). Bars represent 95% CI.

From 2016 through 2018, the incident cases of neuromyelitis optica spectrum disorder (NMOSD) were 11,973 and MS was 9,879 (manuscript submitted for publication) [14]. The ratio of NMOSD to MS among the Chinese was 1•21:1•0. MS has a female predominance, but it is not as pronounced as NMOSD (Fig. 3. B). The female to male ratio is 2•02 (95% CI 1•94–2•11; p < 0•001) for MS and 4•71 (95% CI 4•50–4•94; p < 0•001) for NMOSD.

### 3.3 Burden of hospitalization

The mean age at diagnosis was 45•3 (SD 13•6) years old. Female dominance remains quite stable over the study period, with a proportion about 64%. As the latest government effort to reform China's health care system, the Urban Employee Basic Medical Insurance (UEBMI), Urban Resident Basic Medical Insurance (URBMI) and the New Rural Cooperative Medical Insurance (NRCMI) have dramatically expanded people's access to social health insurance. The UEBMI, URBMI and NRCMI covered about 70% of all patients’ medical payment methods; through the duration of the study, a downward trend was observed in the proportion of fully self-funded patients, from an initial 15•2% to 13•9%. Notably, only 0•4% of MS patients compensated treatment with commercial health insurance. State efforts to negotiate lower prices for disease modifying drugs and shorten duration of hospitalization have reduced the average cost of each hospitalization from US $2509.00 (IQR 1538 - 3893) in 2016 to $2229.00 (IQR 1444 -3661) in 2018 (Table 1), and average length of stay for MS patients shortened from 11 (IQR 7–16) days in 2016 to 10 (IQR 7–15) days in 2018.

**Table 1:** Characteristics of the MS patients in Tertiary Hospital of China, 2016 to 2018.

	2016 (n = 5803)	2017 (n = 5749)	2018 (n = 5973)	Total (n = 15,060)
Demographics				
Age, mean (SD), y	44•7 (15•4)	45•4 (15•8)	45•8 (6•8)	45•3 (13•6)
Children, n (%)	291 (5•0)	287 (5•0)	245 (4•1)	711 (4•7)
Adults, n (%)	5512 (95•0)	5462 (95•0)	5728 (95•9)	14,349 (95•3)
Female, n (%)	3759 (64•8)	3610 (62•8)	3835 (64•2)	9638 (64•0)
Comorbidities, n (%)				
Hypertension	1036 (17•9)	1091 (19)	1189 (19•9)	2832 (18•8)
Diabetes	394 (6•8)	395 (6•9)	489 (8•2)	1084 (7•2)
Hyperlipidemia	723 (12•5)	687 (11•9)	776 (13)	1883 (12•5)
Stroke	803 (13•8)	849 (14•8)	925 (15•5)	2214 (14•7)
Depression/anxiety	204 (3•5)	208 (3•6)	240 (4)	557 (3•7)
Osteoporosis	125 (2•2)	153 (2•7)	160 (2•7)	377 (2•5)
Cancer	60 (1)	74 (1•3)	89 (1•5)	196 (1•3)
Autoimmune diseases	128 (2•2)	134 (2•3)	133 (2•2)	346 (2•3)
Hospital burden				
Number of Hospitalizations, n	9200	8912	9224	27,336
Length of Stay, Median (IQR), days	11•0 (7•0–16•0)	11•0 (7•0–15•0)	10•0 (7•0–15•0)	11•0 (7•0–15•0)
Hospitalization cost, Median (IQR), USD ($)	2509 (1538–3893)	2366 (1483–3699)	2229 (1444–3661)	2368 (1485–3703)
Payment methods, n (%)				
UEBMI	1843(31•8)	1938(33•7)	2132(35•7)	5106 (33•9)
URBMI	892(15•4)	925(16•1)	1259(21•1)	2620 (17•4)
NRCMI	1283 (22•1)	1165 (20•3)	870 (14•6)	2851 (18•9)
CHI	26 (0•4)	22 (0•4)	19 (0•3)	57 (0•4)
Fully self-funded	883 (15•2)	879 (15•3)	831 (13•9)	2228 (14•8)
Other	876 (15•1)	820 (14•3)	862 (14•4)	2198 (14•6)

UEBMI: The Urban Employee Basic Medical Insurance; URBMI: The Urban Resident Basic Medical Insurance; NRCMI: The New Rural Cooperative Medical Insurance; CHI: Commercial Health Insurance; SD: standard deviation; IQR: interquartile range.

### 3.4 Associated comorbidities in patients with MS

The prevalence of risk factors for vascular diseases such as hypertension, diabetes and hyperlipidemia were 18•8%, 7•2%, 12•5%, respectively, in our MS cohort. 2,214/15,060 (14•7%) MS patients experienced a stroke. 557/15,060 (3•7%) patients suffered anxiety and/or depression. 377/15,060 (2•5%) of MS patients developed osteoporosis. Autoimmune conditions were not prevalent amongst patients with MS, with only a proportion of 2•3% in our cohort (Table 1).

### 3.5 Mortality in MS

Hospital mortality among individuals with MS was relatively stable throughout the study period. 104 adults and 2 children died in the period of 2016–2018, the crude mortality rate of MS in hospital was 7•0 deaths per 1000 person-years (95%CI 5•7–8•4). The average death age in this study population was 58•8 (SD 17•5) years old. Most of these patients died either in the neurology department (21•6%) or intensive care unit (21•6%); the latter, tended to have an increased proportion of death. More than half of the deaths were caused by lung infection (24•1%), followed by cardiovascular disease (21•7%), stroke (20•8%), diabetic ketosis (16%), and gastrointestinal bleeding (13•8%) (Table 2).

**Table 2:** Summary of Death.

	2016 (n = 39)	2017 (n = 46)	2018 (n = 31)	total (n = 106)
Demographics				
Age, mean (SD), y	59•6 (16•1)	56•0 (16•2)	62•1 (20•8)	58•8 (17•5)
Female, n (%)	29 (74•4)	23 (50)	18 (58•1)	70 (60•3)
Child/adult	0/39	1/45	1/30	2/104
Department*, n (%)				
Neurology	15 (38•5)	8 (17•4)	2 (6•5)	25 (21•6)
Critical Care Medicine	6 (15•4)	10 (21•7)	9 (29•0)	25 (21•6)
Respiratory	2 (5•1)	3 (6•5)	3 (9•7)	8 (6•9)
Hematology Department	1 (2•6)	1 (2•2)	1 (3•2)	3 (2•6)
Infectious Diseases	1 (2•6)	1 (2•2)	1 (3•2)	3 (2•6)
Causes#, n (%)				
Lung infection	12 (30•8)	9 (19•6)	7 (22•6)	28 (24•1)
Cardiovascular disease	6 (15.4)	9 (19•6)	8 (25.8)	23 (21.7)
Stroke	7 (17.9)	8 (17.4)	7 (22.6)	22 (20.8)
Diabetic ketosis	6 (15.4)	6 (13)	5 (16.1)	17 (16)
Gastrointestinal bleeding	5 (12•8)	7 (15•2)	4 (12•9)	16 (13•8)

⁎ Top 5 departments of discharge,

# The top 5 death related diseases.

## 4 Discussion

This is the first study initiated to estimate the incidence of multiple sclerosis in a population-based health care administrative data-set in mainland China. The age- and sex-adjusted incidence of MS in China is 0•235 per 100,000 person-years, comparable to other East Asian countries. Originally postulated by J.F. Kurtzke [5], geographical distribution of MS risk can be generally distributed in three zones, with North American and northern European countries considered as a high-risk prevalence zone. Asia has been among the low-risk areas, although incidence has been recently increasing [15]. According to the 2013 Atlas of MS launched by the Multiple Sclerosis International Federation, MS prevalence is highest in Middle East and lowest in East Asia [4]. The incidence of MS in Iran reached 6•7/100,000, similar to continental Europe [16]. However, the incidence of MS in East Asian countries is at comparable levels range from 0•2 to 0•8 per 100,000 persons. In the present study, the incidence of MS in Inner Mongolia and Shanxi Province, which are in the same latitude zone as Japan and South Korea, were 0•33 and 0•54 /100,000. The age-standardized incidence of MS was 0•50 per 100,000 in Korea [9], In Japan, the incidence is on the rise from 0•04 in 1980s to 0•78 /100,000 in 2004 [8]. Malaysia, with 23.4% ethnic Chinese, observed a crude annual incidence of 0•55 per 100,000 [10]. In addition to estimating at the national incidence, a map of MS incidence in 31 provinces and municipalities was compiled. We found that the incidence of MS in Shandong Province is 0•29/100,000 for female and 0•12 for male, which is broadly consistent with a hospital-based study on MS incidence in Shandong Province in 2013 [12].

The distribution of incidence rate of MS risk in China where the expansive latitude range and stepwise topography can reveal clues of certain environmental risk-factors. Topographically, the landform of China is low in the east and high in the west; the land surface ascends like a three-step staircase. The plains and lowlands in the east and southeast constitute the first step, occupying about 12 percent of the land. This natural altitude gradient presents an ideal model for interrogating the risk of developing MS and altitude. We find higher incidence rates of MS in high-latitude provinces, such as Inner Mongolia (44° N) and Heilongjiang (47° N). MS incidence is higher in provinces at higher latitudes than those closer to the equator. Additionally, we further confirm that the descending altitude from west to east corresponds with decreased incidence of MS in China. Geographical location is a well-established contributor to the risk of developing multiple sclerosis; [17] a strong latitude gradient was detected for the prevalence of MS, with a 1•03 times increase in prevalence per latitude degree [6]. Moreover, residents living at higher latitude are associated with a younger age of MS onset [18]. Differential exposure to sunlight has been posited as an environmental cause accounting for the positive association between MS incidence and latitude, as ultraviolet (UV) light stimulates the production of vitamin D in the body [17,18]. The link between vitamin D insufficiency and increased risk of MS is well identified [19]. Although higher altitudes generally correspond to increased UV radiation exposure, vitamin D levels do not necessarily increase. A study to compare serum vitamin D levels of plateau Tibetan peoples with Han peoples in Sichuan basin, showed lower serum 25-hydroxy vitamin D in highland Tibetan [20]. Temperature and UV are both factors that stimulate vitamin D production. The climate in the plateau is marked by low temperature. The exposure of the skin is less during outdoor activities in the highland area [21]. Temperature, exposure, diet, and supplements are all confounding factors which obscure vitamin D3s role in manifestation of MS incidence.

A predilection of Asians developing neuromyelitis optica spectrum disorder (NMOSD) has been well recognized. We recently completed a survey for age- and sex-adjusted incidence of NMOSD to be 0•278 per 100,000 person-years (submitted for publication). The annual incident cases of MS to NMOSD in China was 1:1•2. Overall, the ratios of NMOSD to MS are higher in Asia as compared with Western countries [7]. The incidence of NMOSD and MS in Korea was 0•73 and 0•50 per 100,000 persons, respectively [9]. The ratio of NMOSD to MS among the Chinese Malaysians was 2•0:1•0 [10]. A female predominance was observed in the incidence of both NMOSD and MS, but the difference was more pronounced in NMOSD. In our study, the female to male sex ratio of MS and NMOSD was 2•02 and 4•71. The sex ratio of MS varied among different studies with an estimated 3•4:1 in East Asia, following 2•1 in Japan, 1•6 in Korea, 3•2 in Hong Kong, and 3•4 in Taiwan [15]. The female propensity for NMOSD was evident in the Chinese Malaysians rather than for MS by a female to male ratio at 5:1 versus 12:1 in Malaysia [22].

In our study, the peak incidence of MS occurred at the age of 40–49 years. Recently, a shift in peak incidence was found from those aged 40 to 49 years to those aged 30 to 34 years [23]. This is likely attributable to the 2010 McDonald diagnostic criteria for MS, which facilitates earlier diagnosis. Adults developed MS at a later age in China. this could be related to our study method which defines incidence based on new diagnoses rather than time of symptom manifestation, due to the restriction in HQMS information logging.

In our study, prevalent comorbidities are hypertension (18•8%), hyperlipidemia (12•5%), diabetes (7•2) and osteoporosis (2•5%). These are consistent with comorbidities reported in other MS cohorts; hypertension (18•6%) and hyperlipidemia (10•9%) [24]. Few well-designed population-based studies have assessed the incidence or prevalence of autoimmune diseases, cancers, and stroke among patients with MS. Our data report these comorbidities in MS patients as: autoimmune diseases (2•3%), cancers (1•3%), and stroke (14•7%). Depression is the most common associated mental disorder in patients with MS on hospital-based clinics survey [25]. A population-based study evaluated the prevalence of comorbidity at the time of diagnosis in 16,803 Canadian MS patients, wherein a large proportion of patients with MS developed major depression (19•1%) and anxiety (11•1%) [26]. However, the incidence of depression was a relatively low 3•7% in our study. This may be attributed to HQMS only collecting inpatient information and the majority of patients with depression or anxiety are followed up in the clinic. We noticed a high frequency of stroke in MS, in part due to the very high prevalence of stroke in China (1114.8 to 2370/100,000 in China vs 434.86 to 502.32/100,000 the rest of the world) [27,28].

China's basic health insurance, including the Urban Employee Basic Medical Insurance, the Urban Resident Basic Medical Insurance, and the New Rural Cooperative Medical Insurance, covered 70% of MS hospitalization. The burden of hospitalization in MS patients is gradually decreasing by the year. In our cohort, 104 adults and 2 children died, and the hospital mortality rate was 7•0 deaths per 1000 person-years from 2016–2018. The most common causes of death here were respiratory failure (32•8%) and lung infection (24•1%). Several observational studies on MS mortality report inconsistent and competing results. A meta-analysis covering all available mortality studies evaluated the all-cause, cause-specific and gender-specific standard mortality rates. All-cause standard mortality rates per 1000 person-years were 2•56 in males and 3•06 in females [29]. Mortality rates due to infectious diseases and diseases of the respiratory system were higher within the MS population [30].

Our study has several limitations. First, we did not have access to outpatient records. Certain patients with mild symptoms may be diagnosed with clinically isolated syndrome and followed by outpatient visits. The actual incidence may be higher than our current data. Second, results of oligoclonal IgG bands and MRI findings were not collected in this study. Third, we were unable to calculate the prevalence of MS in this study. Prevalence is the product of the incidence rate and the average duration of a condition. The prevalence of MS in China can be well estimated when HQMS contains decades of data in the future.

For the first time, our study captures the incidence for multiple sclerosis across all age groups in almost all Chinese patients. The unique administrative national database enhances the accuracy and rigor of these estimates. The geographical distribution of MS incidence not only correlates to the north-south latitude gradient but also to west-east altitude gradient. Our study fills in the blank of epidemiologic data for approximately 1.4 billion Chinese and enriches the global outlook for this disease. The reported disease burden calls for ramping up regional and global efforts to care for MS patients and investment in research for this devastating and pervasive disease.

## Declaration of Competing Interest

None

## Appendix Supplementary materials

Image, application 1
